# Risk factors and outcomes associated with external ventricular drain infections

**DOI:** 10.1017/ice.2022.23

**Published:** 2022-12

**Authors:** Konrad W. Walek, Owen P. Leary, Rahul Sastry, Wael F. Asaad, Joan M. Walsh, Jean Horoho, Leonard A. Mermel

**Affiliations:** 1 Department of Neurosurgery, Warren Alpert Medical School of Medicine of Brown University, Providence, Rhode Island, USA; 2 Department of Neuroscience, Brown University, Providence, Rhode Island, USA; 3 Norman Prince Neuroscience Institute, Rhode Island Hospital, Providence, Rhode Island, USA; 4 Division of Critical Care, Department of Nursing, Rhode Island Hospital, Providence, Rhode Island, USA; 5 Department of Epidemiology and Infection Control, Rhode Island Hospital, Providence, Rhode Island, USA; 6 Department of Epidemiology and Infection Prevention, Lifespan Hospital System, Providence, Rhode Island, USA; 7 Division of Infectious Diseases, Rhode Island Hospital, Providence, Rhode Island, USA

## Abstract

**Background:**

Insertion of an external ventricular drain (EVD) is a common neurosurgical procedure which may lead to serious complications including infection. Some risk factors associated with EVD infection are well established. Others remain less certain, including specific indications for placement, prior neurosurgery, and prior EVD placement.

**Objective:**

To identify risk factors for EVD infections.

**Methods:**

We reviewed all EVD insertions at our institution from March 2015 through May 2019 following implementation of a standardized infection control protocol for EVD insertion and maintenance. Cox regression was used to identify risk factors for EVD infections.

**Results:**

479 EVDs placed in 409 patients met inclusion criteria, and 9 culture-positive infections were observed during the study period. The risk of infection within 30 days of EVD placement was 2.2% (2.3 infections/1,000 EVD days). Coagulase-negative staphylococci were identified in 6 of the 9 EVD infections). EVD infection led to prolonged length of stay post–EVD-placement (23 days vs 16 days; *P* = .045). Cox regression demonstrated increased infection risk in patients with prior brain surgery associated with cerebrospinal fluid (CSF) diversion (HR, 8.08; 95% CI, 1.7–39.4; *P* = .010), CSF leak around the catheter (HR, 21.0; 95% CI, 7.0–145.1; *P* = .0007), and insertion site dehiscence (HR, 7.53; 95% CI, 1.04–37.1; *P* = .0407). Duration of EVD use >7 days was not associated with infection risk (HR, 0.62; 95% CI, 0.07–5.45; *P* = .669).

**Conclusion:**

Risk factors associated with EVD infection include prior brain surgery, CSF leak, and insertion site dehiscence. We found no significant association between infection risk and duration of EVD placement.

External ventricular drain (EVD) insertion may lead to serious complications including infection, which increases the risk of poor neurosurgical outcomes, increases healthcare costs, and prolongs hospital stays.^1^ The incidence of EVD infection ranges from 0% to 45%^2–21^; the largest study reported that ∼4% of EVDs placed resulted in infection.^4^ Many interventions for reducing infection risk have been proposed. Factors previously associated with increased risk of EVD infection include prolonged EVD drainage, cerebrospinal fluid (CSF) leak, EVD manipulation including frequency of CSF sampling, tract hemorrhage at placement, and insufficient hair clipping.^1–6,8–20,22,23^ Factors associated with reduced risk include perioperative and immediate postoperative antibiotics, as well as antibiotic-impregnated EVDs.^21,24–29^ Some additional risk factors for which there has been no conclusive association with EVD infection include age, sex, Glasgow coma scale (GCS) score, elevated intracranial pressure (ICP) at time of EVD placement, systemic infection, prolonged use of postprocedural antibiotics, prolonged hospital stay prior to EVD placement, involuntary EVD disconnection, placement by a junior surgeon, or coverage of the insertion site with a dressing.^1–6,8–20,22–25^ Controversy continues over whether specific indications for EVD placement (eg, brain trauma or intracranial hemorrhage), history of previous EVD placement, prior neurosurgery, and EVD irrigation contribute to infection risk.^1–6,8–20,22–25^

Several institutions, including our own, have demonstrated a significant reduction in EVD infections through implementation of protocols for EVD insertion and maintenance.^3,6–8,10,11,13–15,17,20,23,30^ In 2007, the Department of Epidemiology and Infection Control at our institution identified a high incidence of EVD infections and, in response, formed an EVD Infection Control Committee. This committee has recorded all EVD infections as they have occurred, has periodically recommended changes in practice, and has assessed the impact of these interventions over time. Major elements of this protocol include minimal EVD manipulation after insertion, no routine CSF sampling, and regular cleansing of the insertion site with alcoholic chlorhexidine while the EVD is in situ.^30^

We undertook a retrospective review of prospectively collected data regarding EVD infections among adult or pediatric patients who received an EVD from March 2015 to May 2019 at our 719-bed, tertiary-care, academic medical center.

## Methods

### Data collection and outcome measures

With approval from our institutional review board (no. 1396435), clinical data were collected from the electronic medical record by one investigator (KW). EVD placement was identified in medical records utilizing billing codes (ie, Current Procedural Terminology–CPT; *International Classification of Disease, Ninth Revision–*ICD-9; and *International Classification of Disease, Tenth Revision* Procedure Coding System–ICD-10-PCS) for procedures associated with ventricular drainage via EVD that occurred during the study period. Individual charts were then reviewed to confirm the placement of an EVD and to exclude patients who had other CSF drainage procedures or miscoded procedures. Inclusion was not limited by age, sex, or indication for EVD placement. We applied the following exclusion criteria: EVD placement as part of treatment for an active CSF infection, EVD dwell time <24 hours, and pregnancy. In total, 479 EVDs placed in 409 patients were included in this study.

Our primary outcome was development of an EVD infection following EVD placement. The Center for Disease Control and Prevention National Healthcare Safety Network (CDC NHSN) has not defined EVD infections. For this study, we used the CDC NHSN definition of meningitis/ventriculitis^31^ in patients with an EVD in situ or within 30 days of EVD removal. Secondary outcomes included EVD duration, need for EVD flushing, CSF leak, dehiscence of surgical site, total number of EVDs placed per patient, 30-day functional outcome via modified Rankin Scale (mRS) score, and total length of hospital stay. Other collected variables included age, sex, GCS at insertion, indication for insertion, comorbidities, history of brain surgery including prior EVD placement, and site of EVD placement. For patients who developed an EVD infection, additional data were collected on microbial culture results, initial and final choice of antimicrobial therapy, antimicrobial treatment duration, and number of days after insertion when EVD infection occurred.

### Treatment, procedural management, and infection control protocol

Indications for EVD placement in this patient cohort included subarachnoid hemorrhage (SAH), intracerebral hemorrhage (ICH), intraventricular hemorrhage (IVH), epidural hematoma (EDH), subdural hematoma (SDH), severe traumatic brain injury (TBI), obstructive hydrocephalus, space-occupying lesion, arteriovenous malformation, or shunt failure. EVD insertions were performed at the bedside or in the operating room. No antibiotic prophylaxis was given to the patient before the procedure or while the EVD was in place. Indications for sampling CSF from an implanted EVD were clinical or imaging suspicion of CNS infection or persistent fever. Routine sampling of CSF was not performed. The insertion site of an indwelling EVD was cleaned daily with alcoholic chlorhexidine and inspected for signs of dehiscence, CSF leak, or infection.

EVDs placed in our institution were predominantly non–antimicrobial-impregnated devices (Codman EDS3, Integra LifeSciences; Princeton, NJ). Antimicrobial-impregnated EVDs were reserved for patients with high suspicion of active CSF infection at the time of EVD placement and were thus excluded from this study.

### Statistical analysis

Data were anonymized and exported to Microsoft Excel (Microsoft, Redmond, WA). Missing variables were assessed, and duplicate entries were removed. Continuous variables with normal and nonnormal distributions were represented as mean (± standard deviation, SD) and median (± interquartile range, IQR) respectively. Categorical data were represented as proportions with percentages. Statistical significance of comparisons between data was determined using the independent sample *t* test between mean values; using the nonparametric Kruskall-Wallis equality-of-populations rank test between median values; and using the χ^2^ test or the Fisher exact test, as appropriate, between categorical data. Cox regression analysis was conducted to determine hazard ratios and contribution of independent risk factors to EVD infection risk. *P* < .05 was considered significant. Data analysis was conducted using Statistical Package for Social Sciences (SPSS) version 24 software (IBM, Armonk, NY) and MedCalc version 19.4 software (MedCalc Software, Belgium).

## Results

**Fig. 1.**
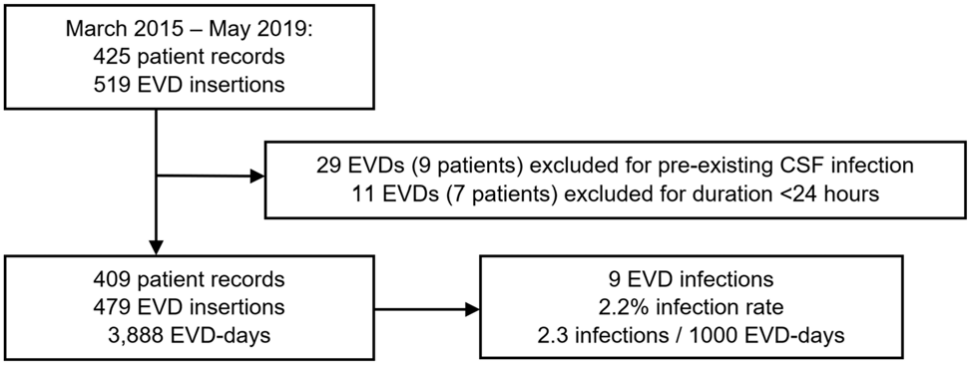
Selection for study Inclusion. Overall, 40 EVD records across 16 patients did not meet the inclusion criteria for analysis due to pre-existing CSF infection (29 EVDs, 9 patients) or EVD duration <24 hours (11 EVDs, 7 patients). Arrows indicate steps in selection process.

Overall, 479 EVDs inserted in 409 patients for 3,888 EVD days were included for analysis (Fig. 1). We observed a similar distribution of sex for patients receiving EVDs, with 55% males, 45% females, an age range of 18 days to 94 years, and a median age of 54 years (Table 1). Most EVD insertions involved patients with SAH (n = 145, 30%), ICH or IVH (n = 112, 23%), and severe TBI including EDH/SDH (n = 110, 23%). At the time of EVD insertion, patients had a median GCS of 7 (IQR, 3–13); 81% were intubated. Moreover, 9 EVD infections occurred in 9 patients, for an overall infection rate of 2.2% (2.3 infections per 1,000 EVD days).

**Table 1.** Patient Demographics

Variable	CSF infection (n=9), No.	No Infection (n=470), No.	*P* Value
Sex, male:female	7:2	256:214	.195
Age at procedure, mean y (SD)	55 (±16)	49 (±22)	.230
GCS at insertion (SD)	8.0 (±4.7)	7.9 (±4.4)	NS
**Age group**			
0–17 y	0	49	NS
18–39 y	2	88	NS
40–69 y	6	272	NS
70+	1	81	NS
**Intubation status**			
Intubated Not intubated	7 2	379 91	NS
**Primary diagnosis**			
SAH	3	142	NS
ICH with IVH	4	108	.282
Severe TBI (including EDH and SDH)	1	109	NS
Obstructive HCP	0	68	NS
Space-occupying lesion	0	21	NS
Arteriovenous malformation	0	12	NS
VP shunt failure	1	10	.205
**Patient immunocompromised?**			
Yes No	0 9	10 460	NS
**History of diabetes?**			
Type I	0	5	NS
Type II	0	35	NS
None	9	430	NS
**History of MRSA/VRE infection or colonization?**			
Yes No	0 9	8 462	NS
**Prior CNS infection?^a^**			
Yes No	0 9	9 461	NS
**Prior brain surgery?**	7	142	.010*
Shunt/EVD only	2	64	.301
TBI ± shunt/EVD	1	19	NS
Space-occupying lesion ± shunt/EVD	1	27	NS
AVM ± shunt/EVD	0	5	NS
Spontaneous hemorrhage ± shunt/EVD	3	27	.012*
**Prior brain surgery with** **concurrent or previous shunt/EVD placement**	5	81	.011*
Shunt/EVD only	2	64	NS
TBI + shunt/EVD	1	6	.136
Space-occupying lesion + shunt/EVD	1	6	.136
AVM + shunt/EVD	0	1	NS
Spontaneous hemorrhage + shunt/EVD	1	4	.100
**Prior brain surgery without** **concurrent or previous shunt/EVD placement**	2	61	NS
TBI	0	13	NS
Space-occupying lesion	0	21	NS
Arteriovenous malformation	0	4	NS
Spontaneous hemorrhage	2	23	.099

Note. SD, standard deviation; NS, not significant; CSF, cerebrospinal fluid; GCS, Glasgow coma scale; SAH, subarachnoid hemorrhage; ICH, intracranial hemorrhage; IVH, intraventricular hemorrhage; TBI, traumatic brain injury; EDH, epidural hemorrhage; SDH, subdural hemorrhage; HCP, hydrocephalus; VP, ventriculo-peritoneal; MRSA, methicillin-resistant *Staphylococcus aureus*; VRE, vancomycin-resistant *Enterococcus* species; CNS, central nervous system; AVM, arteriovenous malformation.

a History of CNS infection, but no active infection at time of EVD insertion.

*P < .05

### Effect of comorbidities on EVD infection risk

In total, 10 EVDs (2.1%) were placed in immunocompromised patients (ie, absolute neutrophil count <500/mm^3^ and/or the use of 1 or more immunosuppressive medications). Also, 5 EVDs (1.0%) were placed in patients with type I diabetes mellitus, and 35 (7.3%) were placed in patients with type II diabetes mellitus. Although the presence of active CNS infection was an exclusion criterion, 9 EVDs (1.9%) were inserted in patients with a history of resolved CNS infection. None of these patients developed an EVD infection (Table 1).

EVDs placed in patients with a history of prior brain surgery was associated with an increased risk for EVD infection (OR, 7.8; 95% CI, 1.6–37.8; *P* = .0097), independent of the specific indication for brain surgery (Table 1). When further stratified by prior brain surgeries with and without concurrent or previous shunt or EVD placement, only surgeries with prior EVD or shunt placement were associated with higher EVD infection risk (OR, 5.7; 95% CI, 1.5–21.7; *P* = .011).

### Effect of operative variables on EVD infection risk

Most EVDs were placed in the neurocritical care unit (NCCU; n = 268, 56%) and emergency department (n = 109, 23%) (Table 2). All 9 EVD infections occurred in the NCCU while the EVD was in place (*P* = .0056). The median EVD day-of-infection diagnosis was 4 days (minimum 2, maximum 11; IQR, 2–7). Most EVDs were placed by neurosurgery residents, most commonly by junior residents (n = 217, 45%). The overwhelming majority of catheters were inserted frontally at the Kocher point (n = 455; 366 right-sided and 89 left-sided). Risk of EVD infection was not influenced by training level of the primary surgeon, anatomic site of EVD insertion, total EVD indwelling time, or number of EVDs per patient.

**Table 2.** Operative Statistics

Variable	CSF Infection (n=9), No.	No Infection (n=470), No.	*P* Value
**Primary surgeon**			
Intern (PGY1)	2	123	NS
Junior trainee (PGY2–3)	4	214	NS
Senior trainee (PGY4–7)	1	80	NS
Consultant or attending physician	2	53	.277
**Anatomic site of EVD**			
** Frontal (Kocher)**	**9**	**455**	NS
** **Right-sided	8	366	NS
** **Left-sided	1	89	NS
** Parietal**	**0**	**11**	NS
** **Right-sided	0	8	NS
** **Left-sided	0	3	NS
** Occipital (Frazier)**	**0**	**4**	NS
** **Right-sided	0	4	NS
**Hospital location where EVD inserted**			
NCCU	9	259	.0056*
Emergency department	0	109	.220
Pediatric ICU	0	40	NS
Trauma ICU	0	31	NS
Operating room	0	26	NS
Medical ICU	0	2	NS
Other	0	3	NS
**EVD duration**			
1–7 d	5	251	NS
8–14 d	3	158	NS
>14 d	1	61	NS
**No. of EVDs per patient** (n=409)			
1	6	301	NS
2	3	72	.217
≥3	0	27	NS
**Did EVD require flushing?**			
Yes	3	104	NS
No	6	366	
**CSF leak from EVD insertion site?**			
Yes	3	8	.0007*
No	6	462	
**Surgical site infection?**			
Yes	1	3	.0734
No	8	467	
**EVD insertion site dehiscence?**			
Yes	1	4	.0908
No	8	466	
**Duration of hospital stay, median d (IQR)**			
Total duration	31 (24–43)	19 (11–29)	.0198*
Duration after EVD placement	23 (19–43)	16 (8–26)	.0449*
**30-day functional outcomes (n=409)**			
Median modified Rankin scale score (IQR)	4 (4–4)	4 (2–6)	NS
Mortality	22% (2/9)	33.3% (133/400)	NS

Note. CSF, cerebrospinal fluid; PGY, postgraduate year; EVD, external ventricular drain; NCCU, neurocritical care unit; ICU, intensive care unit; IQR, interquartile range.

*P* values calculated using χ^2^ goodness of fit or Fisher exact test.

*P < .05.

### EVD infection risk factors

EVD infection was associated with prolonged post–EVD-placement length of stay (23 days vs 16 days; *P* = .045). Upon development of an EVD infection, the device was weaned and removed promptly. If the patient continued to require CSF diversion, it was replaced with an antibiotic-impregnated catheter. Median length of treatment with antibiotics after diagnosis of EVD infection was 14 days (minimum 10, maximum 42; IQR, 14–28). The most common pathogens associated with these infections were coagulase-negative staphylococci (Table 3). Two cases were polymicrobial: one case involving *Micrococcus luteus* and *Pantoea agglomerans*, the other involving coagulase-negative staphylococci and *Cutibacterium acnes*.

**Table 3.** Outcomes of EVD Infections

Age and Sex	EVD Indication	Drainage Duration, Days	Infection Onset, Day	Organism(s)	Antibiotic(s)	Antibiotic Duration, Days	30-Day Outcome, mRS
71M	Shunt failure	2	1	CoNS	Vancomycin	10	6^a^
66M	IVH	6	5	MSSA	Nafcillin	42	4
34M	IVH	11	9	CoNS	Vancomycin	28	3
59M	SAH	10	9	CoNS	Vancomycin	14	4
66M	SAH	2	2	*C. acnes* CoNS	Meropenem	10	4
58M	IVH	6	5	CoNS	Vancomycin	14	4
65F	TBI, SDH	3	3	*C. koseri*	Gentamicin IT Meropenem IV	42	6^a^
25M	IVH	12	11	*P. agglomerans* *M. luteum*	Cefepime, vancomycin	14	4
45M	SAH	18	16	CoNS	Vancomycin	14	3

Note. EVD, external ventricular drain; IVH, intraventricular hemorrhage; SAH, subarachnoid hemorrhage; TBI, traumatic brain injury; SDH, subdural hematoma; CoNS, coagulase-negative staphylococci; MSSA, methicillin-sensitive *Staphylococcus aureus*; *C. acnes*, *Cutibacterium acnes*; *C. koseri, Citrobacter koseri*; *P. agglomerans, Pantoea* agglomerans; *M. luteus, Micrococcus* luteum; IT, intrathecal; IV, intravenous; mRS, modified Rankin Scale score (3=moderate disability, 4=severe disability, 6=death).

a Pateint died.

Cox regression analysis adjusted for age, sex, and indication for EVD placement demonstrated increased infection risk associated with prior brain surgery involving EVD or shunt placement (HR, 8.08; 95% CI, 1.7–39.4; *P* = .010), development of a postoperative CSF leak (HR, 21.0; 95% CI, 7.0–145.1; *P* = .0007), and dehiscence of surgical site (HR, 7.53; 95% CI, 1.04–37.1; *P* = .0407). Duration of EVD placement >7 days was not associated with infection risk (HR, 0.62; 95% CI, 0.07–5.45; *P* = .669).

### Mortality and functional outcomes

In total, 135 (33%) deaths were observed within 30 days after EVD insertion. Of the 274 patients surviving at 30 days following EVD insertion, 48 (18%) were lost to follow-up, 134 (49%) had good functional outcomes (mRS, 0–2), and 92 (34%) had poor functional outcomes (mRS, 3–5). We detected no significant difference in mortality or mRS in patients with EVD infection when compared to those without EVD infection. Of the 2 patients with EVD infection who died, one death was due to complications of infection and the other was due to complications of shunt failure (Table 3).

## Discussion

Previously identified risk factors for EVD infection include prolonged EVD dwell time, prior surgery, CSF leak, frequency of CSF sampling and EVD manipulation, insufficient tunneling of the EVD catheter, tract hemorrhage at placement, and insufficient hair clipping.^1–6,8–20,22,23,25,32–34^ Previous brain surgery has been demonstrated to be an independent risk factor for EVD infection.^2,3,5,6,8,15,16,18,19^ Our findings support these reports. Stratification by type of surgery revealed that this increased risk was specifically related to cranial surgeries with EVD or shunt placement concurrently with or before the surgery. To our knowledge, this has not been previously noted in the literature.

Prolonged EVD duration is associated with an increased risk of EVD-related infection.^2,3,5,9,12–14,16–19,25,32,33^ Surprisingly, we did not find this to be the case. Most infections we observed occurred within 1–7 days of EVD insertion. Prolonged EVD dwell time was associated with a nonsignificant decrease in incidence of EVD infections (2.0% risk for 1–7 days, 1.9% risk for 8–14 days, 1.6% risk for >14 days). We hypothesize that this unexpected result is due to several longstanding, evidence-based infection control protocols in place at our institution, including minimal catheter manipulation after insertion, no routine CSF sampling, and regular cleansing of the insertion site with alcoholic chlorhexidine.^1–3,10,14–20,22,30^

Other known risk factors associated with EVD infection include CSF leak, frequency of CSF sampling, and EVD manipulation.^1,3,5,11,13–15,17–19^ Although our institution has not routinely sampled CSF without suspicion of infection since 2008, analysis of this cohort identified a dramatically increased risk of infection with CSF leak. Indeed, CSF leak was the strongest risk factor identified in our study; although CSF leakage around the insertion site occurred in only 11 EVDs, 3 of these EVDs subsequently became infected. CSF leak has consistently been identified as a major contributor to the risk of EVD infection, and our findings further emphasize the importance of careful surgical technique during EVD catheter placement to ensure watertight wound closure and a tight seal around the catheter to mitigate risk of CSF leak and subsequent EVD infection.

Several prior studies have demonstrated SAH and IVH as independent risk factors for EVD infection.^2,5,9,12,25^ Of 9 EVD infections we identified, 3 occurred in patients with SAH and 4 in patients with ICH or IVH. However, these 2 indications for EVD placement comprised most cases requiring EVD insertion in our study, and the incidende of infection was not significantly higher in either group.

As demonstrated in previous studies, the most common pathogens associated with EVD infections are coagulase-negative staphylococci.^5–8,10–12,14–17,19,22,32,33^ Other common pathogens include *Staphylococcus aureus*, *Cutibacterium acnes*, and *Citrobacter koseri*,^5,7,8,10–12,14–19,22,32,33^ and infections with these pathogens were identified in our study patients.

### Comparison to incidence and mortality in other reported studies

Our overall infection rate was 2.2% and the incidence density was 2.3 infections per 1,000 EVD days which compares favorably with prior studies,^1,2,5–19,22,24,33^ including those that also used the CDC NHSN surveillance definition of meningitis/ventriculitis (Table 4).^5–19^ Additionally, our infection rate is comparable to, and in some cases lower than, institutions using post-EVD antibiotic prophylaxis and antimicrobial-impregnated EVDs.^21,24,26–29,35–39^ Based on a previous analysis of the effects of protocol changes at our institution,^30^ we believe major factors that have contributed to this low infection rate include cutaneous antisepsis with alcoholic chlorhexidine, elimination of routine CSF sampling, and use of a modified tunneling technique with coiling of the catheter under the skin. We have demonstrated that EVD catheters can remain in place without contributing to EVD infection risk as long as meticulously sterile technique is used with minimal device manipulation.

**Table 4.** Incidence and Risk Factors Associated With EVD Infection in Previous Literature

Study	No. of Patients	Infection Rate (%)	Predisposing Risk Factors for EVD Infection
Walek et al, 2021^30^	409	2.2	Prior brain surgery CSF leakage
Kim et al, 2020^5^	247	10.1	Multiple EVD insertions Duration of EVD drainage
Flint et al, 2017^6^	308	0.3	Diabetes mellitus
Talibi et al, 2016^7^	66	6.1	Not using antibiotic-impregnated catheter
Phan et al, 2016^8^	110	11.5	Multiple drains (EVD replacement) Not using perioperative systemic antibiotics Not stopping antibiotics within 24 hours of EVD placement
dos Santos et al, 2016^9^	94	5.3	Duration of EVD drainage >10 days
Kubilay et al, 2013^10^	2928	1.5	Not using prophylactic antibiotics Lack of anti-microbial impregnated catheter
Camacho et al, 2013^11^	178	4.8	Lack of educational intervention Lack of strict adherence to aseptic protocol
Kim et al, 2012^12^	343	3.5	Duration of EVD drainage Concurrent systemic infection
Kitchen et al, 2011^13^	39	10.2	Lack of strict aseptic technique during EVD management Lack of care bundles for EVD management
Hoefnagel et al, 2008^14^	228	23.2	Duration of EVD drainage >11 days CSF sampling frequency
Dasic et al, 2006^15^	59	11.9	EVD insertion outside of operating room Lack of prophylactic antibiotics Insufficient tunneling distance of catheter CSF sampling frequency
Bota et al, 2005^16^	638	9.1	Duration of EVD drainage SAH or IVH Craniotomy Coinfection
Korinek et al, 2004^17^	175	5.7	CSF leakage CSF sampling frequency EVD manipulation Duration of EVD drainage >5 days
Lyke et al, 2001^18^	196	5.6	Duration of EVD drainage CSF leakage
Mayhall et al, 1984^19^	172	11.0	ICH with IVH Prior neurosurgery ICP >20 mmHg Irrigation of EVD

Note. EVD, external ventricular drain; CSF, cerebrospinal fluid; SAH, subarachnoid hemorrhage; IVH, intraventricular hemorrhage; ICH, intracranial hemorrhage; ICP, intracranial pressure.

Of the 9 patients who experienced an EVD infection during our study period, 2 died within 30 days of EVD placement, conferring a 30-day mortality rate of 22%. This finding is consistent with previously reported 30-day mortality rates of 17%–46%.^5,9,11,12,16,19^

This study had several limitations. The retrospective nature of this study leaves open the possibility of incomplete data in the medical records. However, microbiological reports were always available. Routine CSF cell counts and CSF lactate levels were not obtained throughout the course of the study; therefore, no conclusions can be drawn as to their ability to help predict or aid in the diagnosis of CSF infection associated with EVD placement. We also did not collect data on which provider manipulated the EVD once it was placed, and the number of insertion attempts per EVD was not routinely recorded. Our study may have been underpowered to identify all significant risk factors in this cohort.

In conclusion, risk of EVD infection was associated with prior brain surgery associated with EVD or shunt placement, CSF leak, and insertion site dehiscence. We did not find an association between infection risk and prolonged duration of EVD placement, which might be attributable to our institutional policies of minimal EVD manipulation after insertion, no routine CSF sampling, and regular cleansing of the insertion site with alcoholic chlorhexidine.
